# Histological and molecular evaluation of *Mentha arvensis* extract on a polycystic ovary syndrome rat model

**DOI:** 10.5935/1518-0557.20220052

**Published:** 2023

**Authors:** Golnoosh Sharafieh, Fatemeh Salmanifarzaneh, Negin Gharbi, Fatima Masoomi Sarvestani, Fatemeh Rahmanzad, Mehdi Razzaghshoar Razlighi, Azizollah Bakhtari, Nazanin Nazari

**Affiliations:** 1 Department of Clinical Biochemistry, School of Medicine, Tehran University of Medical Sciences, Tehran, Iran; 2 Department of Clinical Biochemistry, Islamic Azad University, Shahr-e-Qods Branch, Qods, Tehran, Iran; 3 Department of Biology, Zarghan Branch, Islamic Azad University, Zarghan, Iran; 4 Clinical Neurology Research Center, Shiraz University of Medical Sciences, Shiraz, Iran; 5 Persian BayanGene Research and Training Center, Shiraz University of Medical Sciences, Shiraz, Iran; 6 Department of immunology, College of Veterinary Medicine, Science and Research Branch, Islamic Azad University, Tehran, Iran; 7 Department of Biology, East Tehran Branch, Islamic Azad University, Tehran, Iran; 8 Reprodutive Biology Department, School of Advanced Medical Sciences and Technologies, Shiraz University of Medical Sciences, Shiraz, Iran; 9 Department of Immunology, Shiraz University of Medical Sciences, Shiraz, Iran

**Keywords:** polycystic ovary syndrome, Mentha, CYP17, Ptgs2

## Abstract

**Objective:**

This study aimed to investigate the impact of Mentha arvensis on a rat model of polycystic ovary syndrome (PCOS).

**Methods:**

The PCOS rat model was made by the daily subcutaneous injection of testosterone enanthate (250mg/kg) for 21 days. Thirty rats were divided into five groups, including a healthy control group and four PCOS groups treated with various concentrations of hydroalcoholic extract of *Mentha arvensis* (0, 50, 100 and 200mg/kg). LH and FSH were measured in the blood. The ovaries were used for histological investigation, Cyp17 and Ptgs2 genes expression and total antioxidant capacity.

**Results:**

Our results indicated that the level of LH and FSH hormones in treated PCOS rats with various concentrations of *M. arvensis* were reduced in comparison with the untreated PCOS group (*p*<0.01). Mentha arvensis in the highest concentration (200mg/kg) decreased the number of cysts in this group in comparison with the untreated PCOS group (*p*<0.01). The expression of *Cyp17* and *Ptgs2* genes in the treated group with the highest concentration of hydroalcoholic extract were decreased in comparison with the untreated PCOS group (*p*<0.05). Moreover, the antioxidant capacity in the rats receiving *Mentha arvensis* hydroalcoholic extract was significantly increased in comparison with that from the untreated PCOS rats (*p*<0.05).

**Conclusions:**

For the first time, *Mentha arvensis* hydroalcoholic extract proved to reduce some polycystic ovary syndrome symptoms. In the present experiment, a dose of 200mg/kg of *Mentha arvensis* hydroalcoholic extract was regarded as the most efficient dose.

## INTRODUCTION

Polycystic ovary syndrome (PCOS), one of the most common endocrine system disorders, affects women during their reproductive age (McCartney & Marshall, 2016). This disease was described in 1935 by Stein and Leventhal, that is why it is also named Stein-Leventhal syndrome (Leventhal, 1958). In this disease, approximately 10 small cysts with diameters from 2 to 8 mm are formed in one or both ovaries; or the ovarian volume in at least one ovary is more than 10 ml (Jonard *et al*., 2003). In healthy women, immature oocytes are matured under the effect of Follicle-stimulating hormone (FSH) and ovulation, as well as final maturation is induced by Luteinizing Hormone (LH). While, in women with PCOS, gonadotropin-releasing hormone (GnRH) is increased, and subsequently LH is promoted in comparison with FSH in circulation (Banaszewska *et al*., 2003).

Since androgen hormones are greatly produced in PCOS, the expression of involved genes is changed in the production of endogenous hormones. One of these genes is the Cytochrome P450-C17 gene (*Cyp17*), which encodes the cytochrome P40 enzyme. It is involved in the production of 17-hydroxypregnenolone and 17-hydroxyprogesterone from pregnenolone and progesterone, respectively (Gilep *et al.*, 2011). *Ptgs2* or *Cox2* gene are important in ovulation. They are involved in the production of prostaglandin as an inflammatory dilator in the ovary (Fox *et al*., 2019).

Multiple studies have indicated that oxidative stress, a mismatch between oxidation and antioxidation, is one of the causes of PCOS and PCOS-related symptoms, such as insulin resistance, hyperandrogenic and chronic inflammation (Zuo *et al*., 2016). In view of the fact that epidemiological findings showed a high prevalence of PCOS worldwide (Yildiz *et al*., 2012), PCOS increases the risk of many diseases, such as infertility (Hart, 2008), cancer (Balen, 2001), type II diabetes mellitus (Elting *et al*., 2001), obesity (Elting *et al*., 2001) and heart disease (Dokras, 2013) in these women. Thus, this disease affects all aspects of a woman’s mental and physical health, and subsequently it will have negative mental and economic impacts on family health.

Regarding the side effects of chemical drugs on PCOS treatment, identification and production of alternative drugs are conducive to improve PCOS and its symptoms. Medicinal plants were applied for treating a high range of gynecological diseases, including menopausal and menstrual symptoms. *Mentha arvensis* is a species of the Lamiaceae family of plants, which can grow in temperate wet climates and even in humid places with steppe climates (Ferreira *et al*., 2012). Various species of the Lamiaceae family have been used to treat various women’s health problems, like dysmenorrhea (Bafor *et al*., 2021; Uritu *et al*., 2018), amenorrhea (Elahi *et al*., 2016; Moini Jazani *et al*., 2018) and PCOS (Abasian *et al*., 2018; Goswami *et al*., 2012). But no study has been conducted to investigate the effects of *M. arvensis* on PCOS. Regarding the high prevalence of PCOS and complications, and failure of chemical treatments in these patients, we investigated the impact of *M. arvensis* hydroalcoholic extract on LH and FSH hormones in blood, as well as the expression of *Cyp17* and *Ptgs2* genes and antioxidant capacity of ovary in a PCOS rat model.

## MATERIALS AND METHODS

### Plant collection and extraction

For this experimental study, *M. arvensis* was collected from the province ofFars, Iran. It was identified by a botanist; it was given a voucher number and it was deposited in the Shiraz School of Pharmacy Herbarium.

The plant was dried with all its organs, and it was grinded. To provide hydroethanolic extract, 100 g of dried plant was soaked in 700 ml 80% ethanol and the mixture was shaken for 48 hours, and it was repeated twice. The solution was filtered and the extracts were condensed by a rotary device at 40-50°C for two days. The provided powder was stored at 4°C until the assays were performed.

### Animals

Thirty female wistar rats (6-7 weeks old), weighting 195±4.1 were provided by the Pasture Iran institute animal house. The animals were randomly divided into five groups of six rats each, including a healthy control group, untreated PCOS group (PCOS), PCOS rats treated with 50 mg/kg (T50 mg/kg), 100 mg/kg (T100 mg/kg) and 200 mg/kg (T200 mg/kg) hydroalcoholic extract of *M. arvensis*. The rats were maintained into 23±3°C with free access to food and water in 12 h light: 12 h dark cycles.

Furthermore, the treatment groups of 50, 100 and 200 mg/kg of extract powder mixed with water for 4 weeks were fed by gavage at a volume of 0.5 ml. The weight of all rats was measured before the treatment, after PCOS induction and the last day of experiment. All animal care and procedures in this study were approved by the Ethics committee of the Islamic Azad University, Shahr-e-Qods Branch (No. IAUSG 1926).

### Polycystic ovary syndrome rat model

Rats in the PCOS group, were daily administered 250 mg/kg of testosterone enanthate (Aburaihan Pharmaceutical Co., Iran) by subcutaneous injection in the neck for 3 weeks. After that, ovarian tissue and the number of follicles were examined to confirm the PCOS induction.

### Measurement of LH and FSH hormone levels

After four weeks of *M. arvensis* extract treatment, blood samples were harvested from the rats’ hearts. To prepare the serum, blood samples were centrifuged at 2500rpm for 10 minutes and levels of LH and FSH hormones (ZellBio GmbH, Germany) were assessed using the ELISA technique, based on instructions from the manufacturer, by ELISA Reader at a wavelength of 450nm.

### Ovarian morphology

In the end of the experiment, the ovaries of all groups were stained for histological assessment by hematoxylin and eosin (H&E). Briefly, after dissecting the ovaries, the samples were fixed in formaldehyde. Then, the routine paraffin embedding was done. The ovary tissues were cut using a rotary microtome at a thickness of 5 µm. The samples were stained with H&E and examined under a light microscope (Nikon ECLIPSE E200, Japan).

### Total antioxidant capacity (TAC) in ovarian tissues

The ovaries were removed and homogenized in PBS buffer, and then centrifuged at 12,000 rpm for 15 minutes at 4°C. After that, the supernatant was transferred into a new tube and TAC was measured using the Kiazist TAC kit (Kiazist, Iran) according to instructions from the manufacturer. Absorbance was read at 450 nm wavelength.

### The assessment of *Cyp17* and *Ptgs2* gene expression

Real time RT-PCR was used to evaluate the expression of *Cyp17* and *Ptgs2* (*Cox2*) genes in ovarian tissue samples. To extract RNA, we used the RiboEX kit (GeneAll, Seoul, South Korea), according to instructions of manufacturer. A nanodrop device was used to determine the amount and purity of the extracted RNA. In the next step, cDNA was synthesized using a HyperScript™ first strand cDNA synthesis kit. Real-time RT-PCR was performed using an Applied Biosystems StepOnePlus™ (Applied Biosystems™, Foster City, CA, USA) in a final volume of 25 µL containing of 1 µl of the cDNA template, 12.5 µL of RealQ Plus 2x Master Mix Green (Amplicon, Denmark), and 1 µL (10 pmol/µl) of each specific primer (Table 1). Glyceraldehyde-3-phosphate dehydrogenase was used as a reference gene. The samples were analyzed using the 2^-∆∆Ct^ method.

**Table 1:** Specific primer characteristics of Cyp17 and Ptgs2 and Gapdh genes.

Gene	Oligonucleotide sequence (‘3-5’)	Tm (◦C)	Product length
*Gapdh*	Forward: AAGCTGGTCATCAACGGGA	60	180
	Reverse: GAAGGGGCGGAGATGATGAC		
*Ptgs2*	Forward: GATGACGAGCGACTGTTCCA	60	223
	Reverse: CAATGTTGAAGGTGTCCGGC		
*Cyp17*	Forward: GACTGTGACCTGGGAAGTGATA	60	214
	Reverse: GGCGTCTTTGACTTGACCCA		

### Statistical analysis

Data analysis was performed by GraphPad Prism version 6 (GraphPad Software). One-way ANOVA test was applied to check data significance. Tukey’s multiple comparison was used as a posthoc to compare the groups. All results were presented as mean ± standard error (mean±SEM). We considered a significance level of 0.05. we used five replicates per group for each assessment.

## RESULTS

### Body weight

The rats’ weights were measured before starting the study, after creating the PCOS model and on the last day of the study (Table 2). In the last day of the experiment, the body weight in the PCOS group was higher than among controls and the T50, T100 and T200 groups (*p*<0.05). After 21 days of treatment with hydroalcoholic extract of *M. arvensis*, body weight of rats in the T50, T100 and T200 groups reached the levels of the control group.

**Table 2:** Effects of *Mentha arvensis* extract on body weight during three stages of the experiment.

	Control	PCOS	T50	T100	T200
First day	195±3.3	194±3.5	191±4.2	192±2.7	189±4.1
After PCOS induction	202±3.1	199±1.8	199±2.3	202±2.8	201±3.1
Last day	219±2.8^#^	229±2.1	218±2.6^#^	217±1.8^#^	212±2.2^#^

T200, T100, T50: Treated PCOS group with 50, 100 and 200 mg hydroalcoholic extract of *M. arvensis*, respectively. Data are presented as mean±SEM.

Shows a significant difference with PCOS group at *p*<0.05.

### LH and FSH hormones

**Figure 1:**
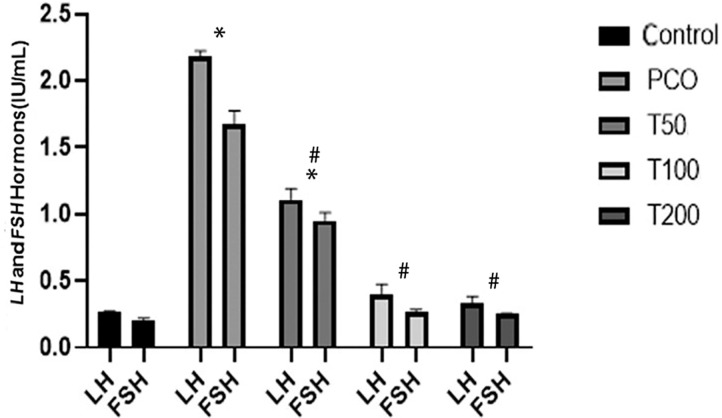
The changes in LH and FSH hormones in the rat groups: LH and FSH hormones (IU/mL) in the healthy control group, untreated PCOS group (PCOS), treated groups with 50 mg (T50), 100 mg (T100) and 200 mg (T200) hydroalcoholic extract of *M. arvensis*. * Shows a significant difference with the control group at *p*<0.001, and # Shows a significant difference vis-à-vis the PCOS group at *p*<0.01.

The levels of LH and FSH hormones in the serum of rat groups are presented in Fig. 1. LH and FSH levels in untreated PCOS and T50 groups significantly increased in comparison with the healthy controls, the T100 and T200 groups (*p*<0.01). It was also found that the levels of LH and FSH in the PCOS group were higher than theT50 group (*p*<0.01). On the other hand, levels of LH and FSH hormones in the T100 and T200 groups reached the values of the control group (Fig. 1).

### Ovarian morphology

**Figure 2:**
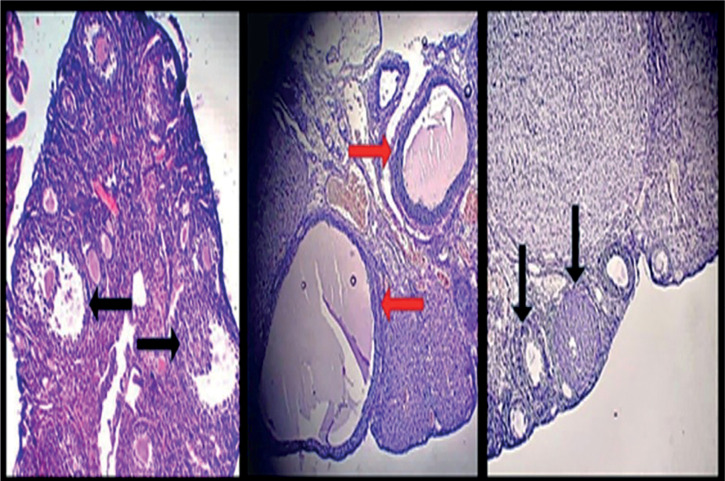
Ovarian cross section photomicrograph (H&E) of control, PCOS and treated groups with 200 mg/kg hydroalcoholic extract of *M. arvensis* (T200 mg/Kg) with 400x magnification. Red arrows show the cysts in the PCOS group (middle image), and the black arrows represent a healthy follicle in the ovaries of control group (left image) and T200 group (right image) animals.

Ovarian tissues of controls, PCOS and treated group (with 200 mg/kg hydroalcoholic extract of *M. arvensis*) were assessed using hematoxylin and eosin staining (Fig. 2) with a magnification of 400s. As indicated in Fig. 2 in the control group, healthy follicles were visible with black arrows and in the PCOS group cysts were indicated with red arrows. In the T200 group, healthy follicles are represented by a black arrow.

### Number of follicles

We counted the number of follicles in the ovaries from three rats of each group. Results showed that the number of ovarian follicles in the PCOS group (5±1.3) was lower than that in healthy rats (23±1.4. *p*<0.01). This number was increased in PCOS rats treated with *M. arvensis* hydroalcoholic extract (*p*<0.01); and the highest number of follicles was observed in the T200 group (21±2.6), which was not significantly different from the number of follicles among healthy controls Table 3.

**Table 3:** The number of follicles in the ovaries after treating the PCOS rats with different concentration of hydroalcoholic extract of M. arvensis.

	Control	PCOS	T50	T100	T200
The number of follicles	23±1.4	5±1.3^*^	11±2.5^*#^	17±1.7^*#^	21±2.6

T200, T100, T50: Treated PCOS group with 50, 100 and 200 mg hydroalcoholic extract of M. arvensis, respectively. Data are presented as mean±SEM.

* Shows a significant difference with control group at *p*<0.01, and # Shows a significant difference with PCOS and T50 groups at *p*<0.01.

### Total antioxidant capacity of ovarian tissue

**Figure 3:**
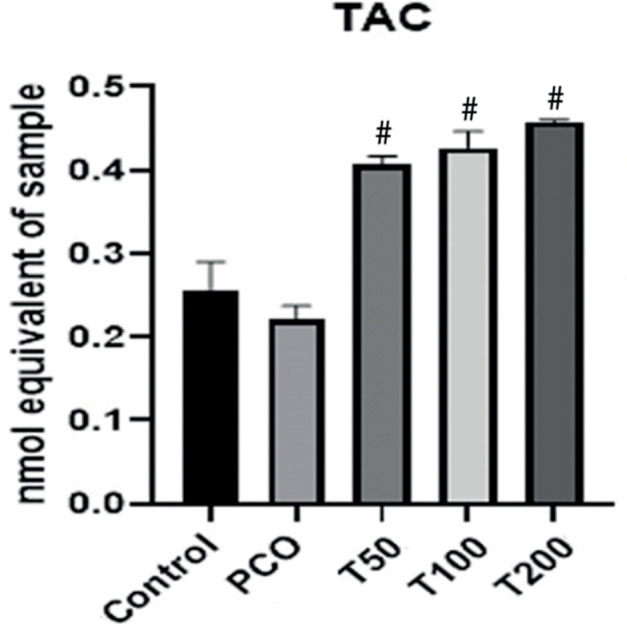
Total antioxidant capacity of ovarian tissues. Total antioxidant capacity was measured in the control, untreated PCOS (PCOS), treated PCOS group with 50 mg (T50), 100 mg (T100) and 200 mg (T200) hydroalcoholic extract of *M. arvensis*. # Shows a significant difference in the PCOS group at *p*<0.05.

This finding indicated that the total antioxidant capacity in the PCOS group was significantly lower than the PCOS-treated groups with different concentrations of *M. arvensis* extract (T50, T100 and T200, *p*<0.05). There were no significant differences among the treated groups (Fig. 3).

### *Cyp17* and *Ptgs2* gene expression in the ovaries

**Figure 4:**
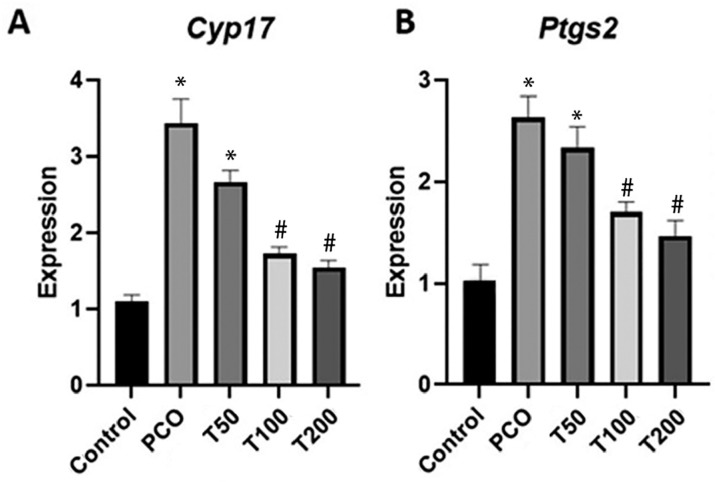
The expressions of *Cyp17* and *Ptgs2* genes in ovarian tissue. Expression of *Cyp17* (4A) and *Ptgs2* (4B) genes in ovarian tissue of healthy controls, untreated PCOS (PCOS), treated group with 50 mg (T50), 100 mg (T100) and 200 mg (T200) hydroalcoholic extract of M. arvensis. * Shows a significant difference vis-a-vis control group at *p*<0.01, and # Shows a significant difference with PCOS and T50 groups at *p*<0.01.

Real time RT-PCR results indicated that the *Cyp17* and *Ptgs2* gene expressions in ovarian samples of PCOS and T50 groups were significantly enhanced in comparison with the control group (Fig. 4, *p*<0.01). Furthermore, expressions of these genes were significantly reduced in the T100 and T200 treated groups in comparison with the PCOS group (*p*<0.05).

## DISCUSSION

PCOS is a common metabolic disorder in women of childbearing age, which affects all aspects of a woman’s life. The criteria for diagnosis of this disease are LH and FSH hormone level as well as ovarian histological changes and weight gain of patients. In this study, we approved creating a rat model using features like rat weight, LH and FSH hormones and ovarian tissue changes.

In the present study we found that hydroalcoholic extract of *M. arvensis* in all concentrations inhibited weight gain in the PCOS rats; although the concentration of 200 mg/kg had the highest impact on body weight improvement. Alizadeh *et al*. (2020) evaluated the effects of hydroalcoholic extract of *Stachys sylvatica* from Lamiacea family on the inhibition of obesity in the PCOS rat model. Their study indicated that the extract improved obesity in PCOS rats.

In healthy women, the level of the two hormones FSH and LH were almost the same (Swaroop *et al*., 2015); but in PCOS women the LH level was increased. The results of our study showed an increase in LH in the PCOS group. The groups treated with 100 and 200 mg/kg of hydroalcoholic extract of *M. arvensis* significantly decreased the LH and FSH to their levels in healthy rats. Mentha species, like *M. arvensis* have high amounts of phenolic compounds as secondary metabolites (Benabdallah *et al*., 2016; Gharib & da Silva, 2013), and the most significant phenolic compounds in these species are flavonoids (Haj-Husein *et al*., 2016). Some flavonoids can activate GABA by binding to its receptor (Ciechanowska *et al*., 2009). Gonadotropins stimulate the secretion of LH and FSH hormones from the pituitary gland. On the other hand, gonadotropin secretion is prevented by GABA neurotransmitter (Ciechanowska *et al*., 2009). Thus, by binding the flavonoids to GABA receptors, GABA prevents the GnRH secretion and subsequently, the level of LH secretion will be reduced (Herbison & Dyer, 1991). At variance with those results, Alizadeh *et al*. (2020) study indicated that *Stachys sylvatica* hydroalcoholic extract significantly reduced the levels of FSH and LH in treated rats. Also, Grant’s (2010) study showed that spearmint (Lamiacea family) significantly increased LH and FSH in women with PCOS. The results of another study indicated that Marjoram (Lamiacea family) as tea, had no change in the level of LH hormone in women with PCOS (Haj-Husein *et al*., 2016). These differences in the studies could be attributed to variances in the PCOS induction drug, the investigated subject, and the type of plant extract used.

This study showed a significant increase in the number of cystic follicles in the PCOS group in comparison with the healthy control group. The number of ovarian cysts was reduced after treatment with *M. arvensis* hydroalcoholic extract. These findings are consistent with the results of some other species of Lamiacea family and other herbal extracts in the treatment of PCOS (Alizadeh *et al*., 2020; Amoura *et al*., 2015; Sadeghi Ataabadi *et al*., 2017).

Our findings indicated an important increase in the antioxidant capacity of the ovary of PCOS rats treated with *M. arvensis* hydroalcoholic extract. The research of Sadeghi Ataabadi *et al*. (2017) indicated that *Mentha spicata* extract in PCOS rats decreased ovarian cysts and increased ovarian antioxidant capacity. Increasing oxidative stress by an imbalance between antioxidants and oxidants, which can disrupt the folliculogenesis in PCOS patients and consumption of antioxidant substances can be efficient in the treatment of PCOS (Zhang *et al*., 2008). Multiple studies showed that the increase in antioxidant capacity of Mentha species such as *arvensis* is due to its phenolic compounds (Benabdallah *et al*., 2016; Dar *et al*., 2014).

In this study, the expression of *Cyp17* in ovarian tissue of PCOS rats was considerably high. In agreement with this finding, many studies revealed that the expression of *Cyp17* in PCOS ovarian tissue of humans and rats was greatly expressed compared to healthy ovarian tissue (Li *et al*., 2013; Nelson *et al*., 1999; Wickenheisser *et al*., 2005). This study for the first time presented that *M. arvensis* hydroalcoholic extract considerably decreased the expression of *Cyp17* in PCOS rats. Increasing *Cyp17* production increases androgen production. Subsequently, misplaced androgen production plays a pivotal role in the PCOS etiology (Rosenfield & Ehrmann, 2016). Hence, reduction of PCOS symptoms in treated rats with *M. arvensis* extract can be due to the influence of this plant on downregulation of the *Cyp17* expression and subsequently, reduction of androgen production.

The expression of *Ptgs2* gene as a follicular phase marker was considerably high in the ovarian tissue of PCOS rats. Several studies reported high expression of this gene in PCOS patients (Liu *et al*., 2016; Schmidt *et al*., 2014). One of the mechanisms of pathogenesis suggested that gene expression of zinc finger gene 217 (*Znf217*) was decreased in PCOS rats and women. This gene increases the expression of inflammatory genes, such as *Ptgs2* and Prostaglandin E2 (*Pge2*) (Zhai *et al*., 2020).

## CONCLUSION

This study confirmed that *M. arvensis* hydroalcoholic extract can be useful as a treatment for PCOS patients by improving follicularization and regulating LH and FSH secretion. It seems that the antioxidant features of *arvensis* extract are beneficial in reducing the symptoms of this disease, and it is suggested that different extract compounds, especially phenolic compounds, play an important role in this activity.
